# The effects of executive coaching on behaviors, attitudes, and personal characteristics: a meta-analysis of randomized control trial studies

**DOI:** 10.3389/fpsyg.2023.1089797

**Published:** 2023-06-02

**Authors:** Andreea Nicolau, Octav Sorin Candel, Ticu Constantin, Ad Kleingeld

**Affiliations:** ^1^Faculty of Psychology and Education Sciences, Alexandru Ioan Cuza University, Iaşi, Romania; ^2^Department of Industrial Engineering and Innovation Sciences, Eindhoven University of Technology, Eindhoven, Netherlands

**Keywords:** executive coaching, coaching outcomes, coaching effectiveness, organizational performance, meta-analysis

## Abstract

**Background:**

A growing number of studies emphasize executive coaching as an effective developmental tool that managers can use to increase their performance in organizational settings. However, the coaching research suggests a large variety of processes and outcomes, lacking clarity on the primary psychological dimensions most impacted.

**Method:**

Reviewing 20 studies with a rigorous methodological design that used control trials and pre-post tests, we evaluated and compared the relative effects of coaching on different types and sub-types of outcomes by means of a classification of coaching outcomes based on previously used taxonomies.

**Results:**

The results indicate that the impact of coaching on behavioral outcomes was higher compared to attitudes and person characteristics outcomes, suggesting that behavioral coaching outcomes, especially cognitive behavioral activities, are the most impacted by executive coaching. Moreover, we found significant positive effects for some specific outcomes, such as self-efficacy, psychological capital, and resilience, indicating that executive coaching is effective in producing change even on dimensions considered relatively stable over time. The results show no moderation effects of the number of sessions. The length of the coaching program was a significant moderator only for the attitudes outcomes.

**Discussion:**

These findings provide evidence that executive coaching is a powerful instrument for organizations to support positive change and personal development.

## Introduction

Rapid changes in and the uncertainty of the economic, political, technological, and social environment require leaders to develop human capital and the capacity to respond to these challenges and achieve strategic organizational goals (Kim et al., 2014). This complex context increases the demands and expectations on leadership development to cultivate leaders who can maintain and create optimal conditions for employees and long-term outcomes. Executive coaching is now considered one of the primary methodologies for supporting leaders' development (MacKie, 2014). Executive coaching concerns the supported changes in individuals with managerial authority and responsibility in an organization to enhance their professional performance and personal aspirations (Kilburg, 1996). Organizations widely use coaching to create cultural and wellbeing change (O'Connor and Cavanagh, 2013). It enables long-term solutions by developing new habits, attitudes, and work practices through a systemic approach (Ballesteros-Sánchez et al., 2019). Its increased popularity has led to significant financial investment and over 71,000 coach practitioners worldwide (ICF, 2020).

Despite the high demand for executive coaching and the increased interest from scholars, there is significant debate about what outcomes can be achieved and where coaching resources should be targeted (MacKie, 2014). Executive coaching outcomes for an individual or organization can be manifold because they differ depending on the client's goal and the social context (Athanasopoulou and Dopson, 2018). Thus, how executive coaching outcomes are defined and classified varies from one study to another, making it difficult to assess what is most impacted. One of the main limitations of the past research in evaluating coaching effectiveness centers on choosing from various outcomes and their benefits (Leonard-Cross, 2010; Graßmann et al., 2020). Therefore, previous meta-analyses pointed out the lack of a clear conceptual framework for categorizing coaching outcomes and the importance of having one (Theeboom et al., 2014). Although some criteria for classifying coaching outcomes have been proposed and analyzed in previous meta-analyses, they only have been defined based on the criteria measured and applicable to the included studies. Grant (2010), Theeboom et al. (2014) suggested that a robust framework incorporating theoretical perspectives from several sub-disciplines of psychology could enrich the coaching literature and benefit practitioners in improving the coaching intervention. We believe that consistency in the definition and use of outcome measures is paramount to obtaining robust insights into the effectiveness of executive coaching. Therefore, we used a systematic framework for coaching outcome classification based on existing typologies in applied psychology research to address the past literature limitations and help evaluate the relative strength of executive coaching on different sub-types of outcomes.

Apart from the difficulty in structuring the outcomes, another challenge for consensus on evaluating the effectiveness of executive coaching is the appropriate research designs (De Meuse et al., 2009; Theeboom et al., 2014; Sonesh et al., 2015; Jones et al., 2016). Most studies on executive coaching investigated the effectiveness of coaching by utilizing a within-subject research design (Jones et al., 2016). In within-subjects designs, the effect sizes represent the differences between measurements before and after the coaching, with time variation between measurements depending on the number of coaching sessions. An alternative design is a between-subjects design where effect sizes represent the differences between the control and the experimental (i.e., coaching) groups (Neal et al., 2006; Orvis et al., 2009). The previous meta-analyses of executive coaching effectiveness (Theeboom et al., 2014; Sonesh et al., 2015; Jones et al., 2016) were based on study selections that include various research designs, such as randomized control trials (RCT) but also quasi-experimental designs that may have overestimated the effect sizes. Theeboom et al. (2014) reported that the studies using a within-subject design show larger effect sizes than those using an independent-group design, implying that the study design considerably influences the relationship between the coaching intervention and the reported outcomes. These conclusions were supported by the findings of a recent meta-analysis using only randomized control trials (Burt and Talati, 2017). Their analysis yielded notably lower overall weighted effect sizes for all coaching outcomes in the small to medium range, compared with a medium to large effect size (Theeboom et al., 2014) and a very large effect size (De Meuse et al., 2009), which was attributed to the difference in methodologies. To our knowledge, just one meta-analysis on executive coaching used only RCT (11 studies; Burt and Talati, 2017). However, several RCT studies on executive coaching have been published since its publication. Therefore, we believe that there is a need for a new meta-analytic study, including exclusively RCT studies on executive coaching, where effects are always established compared to a control group in the same circumstances, and source and selection biases are substantially reduced. Thus, to assess the effectiveness of executive coaching, we have included 20 RCT studies in the current meta-analysis, with only four of these included in the most recent published meta-analysis (Burt and Talati, 2017).

Executive coaching is a supportive relationship with clearly defined individual anticipated outcomes. However, there are questions about how many sessions are needed or how long a coaching program should take. As this study will show, a meta-analysis could investigate and provide evidence on how important these aspects are for the perceived coaching effectiveness on different types of outcomes.

Thus, the present study aims to evaluate executive coaching effectiveness and assess the relative strength of coaching on different types of outcomes by reviewing and analyzing almost 20 years (2006–2023) of published research using the most rigorous research design. Such analyses allow us to inform the coaching buyers' decisions and allow important discussion of coaching effectiveness regarding what is most impacted to facilitate more tailor-made and customized individual and organizational development in the current world context (LeBlanc, 2018). To our knowledge, our study is the first meta-analysis of the relative strength of coaching among different types of outcomes. The present article differs from previous meta-analyses in that our focus is on including only RCT studies and comparing different types of coaching outcomes to understand the most impacted ones rather than including studies using different research designs and merely assessing the overall effect of coaching effectiveness. Our meta-analysis of RCT studies aims to (a) provide a way to structure and analyze the coaching outcomes, (b) examine the effects of executive coaching on the overall outcomes studied and compare its relative strengths on different types of coaching outcomes and (c) analyze possible conditions for coaching effectiveness. Our hope is that our results contribute to improving the way of structuring and analyzing the coaching outcomes and the decisions on how to allocate coaching resources and support.

## Theoretical framework

### Defining executive coaching

Executive coaching—also referred to as business coaching, leadership coaching, or workplace coaching—is an individualized learning and development intervention to help leaders improve their capabilities, enhance their effectiveness and maximize their personal and professional potential in their organizations (Richardson, 2010; Jones et al., 2016; Williams and Lowman, 2018).

Executive coaching is considered a new discipline aimed at enhancing leaders' growth and development (Finn, 2007; Moen and Skaalvik, 2009). In organizational settings, executive coaching is a means for executives to increase knowledge, improve competencies, and enhance effective leadership behavior favoring the company (Elliott, 2011; Moen and Federici, 2012). It has been suggested that executive coaching developed exponentially in the 1980's. One of the reasons was the volatile economic context, which required leadership development and outplacement, among other needs (Richardson, 2010).

Kilburg (1996) and Grant et al. (2009) defined executive coaching as a helping, collaborative relationship between a client who has managerial authority and responsibility in an organization and a professional coach, which focuses on setting mutually defined goals and actions with the aim of improving their professional performance and personal aspirations, and, consequently, the effectiveness of the organization. An executive coach facilitates continuous learning, provides emotional support and feedback, and helps managers to achieve a performance breakthrough and a fundamental transformation (Evers et al., 2006; Finn, 2007; Fontes and Dello Russo, 2021).

Despite various approaches and models being available and used, common features of executive coaching interventions include goal-setting, awareness-raising, commitment and accountability, planning, and action (Grant et al., 2009; Jones et al., 2016). A particularity of executive coaching is its focus on goal-directed initiatives (Grant and Cavanagh, 2007; Spence and Oades, 2011). It is primarily concerned with setting goals related to strategic organizational objectives and improving performance at work (Moen and Skaalvik, 2009; Athanasopoulou and Dopson, 2018).

### Previous meta-analyses

De Meuse et al. (2009) conducted the first review of studies on executive coaching outcomes and assessed its effectiveness using a meta-analytic approach. Only six of the 12 studies used external coaches and a methodological design that provided pre- and post-data. Their findings point to positive effects of executive coaching, ranging from improved individual skills and behaviors to increased team performance. However, their meta-analysis found a lack of consistency in the impact of executive coaching due to the specific content area being coached. They suggested that the outcomes may vary based on the context and that there may even be negative outcomes in some situations.

Theeboom et al. (2014), in a subsequent meta-analysis, highlighted the need for defining relevant outcomes dimensions for executive coaching. They reported that executive coaching has significant positive effects on five outcomes suggested to be theoretically and practically relevant, which can fit under three main branches: cognitive, affective, and behavioral (Fontes and Dello Russo, 2021). Nevertheless, the large effect size they found may have been partly due to the inclusion of different research designs and the shortage of rigorous studies available. For this reason, the same outcomes—performance, wellbeing, attitude, coping, and goal attainment—were investigated in a meta-analysis conducted by Burt and Talati (2017) using only randomized control trial studies. It should be noted that, even though the authors refer to executive coaching in their meta-analysis, also studies that used life coaching were included, such as studies conducted by Green et al. (2006) and Spence and Grant (2007).

Another framework for executive coaching outcomes was proposed and examined meta-analytically by Jones et al. (2016). Their meta-analysis, which included studies using different research designs, indicated that coaching positively affects skill-based, affective, and individual-level outcomes, with a larger effect size (*d* = 0.5) for individual-level results. Sonesh et al. (2015) conducted a meta-analysis that explored a different three-dimension framework, consisting of relationship outcomes, coachee outcomes, and organizational outcomes. Their findings suggested that coaching is an effective development tool that contributes especially to improving the coachee outcome related to the behavioral change dimension. Although both meta-analyses reported positive effects of coaching, the results have likely been influenced by the inclusion of correlational design studies (Sonesh et al., 2015) and studies that used both internal and external coaches (Jones et al., 2016). Internal coaches are employees of the organization who provide coaching to other employees with whom they do not have formal authority. In contrast, external coaches are engaged by the organization based on a contractual agreement for specific coaching hours with specific employees.

### Present study

To evaluate the relative strength of executive coaching on different types of outcomes—the key aim of our study—we propose a classification based on the taxonomy that Bosco et al. (2015) developed. This hierarchical variable taxonomy allows to code and analyze the effect sizes (ES) to provide results on the extent to which Cohen's ES benchmarks reflect the omnibus distribution of findings commonly investigated in applied psychology research and which benchmarks vary across bivariate relation types (e.g., attitude–intention vs. attitude–behavior). The taxonomy resulted from a content-coding scheme created inductively and covers all major areas of industrial–organizational psychology, organizational behavior, and human resource management. In total, the taxonomy includes 4,869 levels nodes (i.e., variable names or category names), 11 first-level nodes (person characteristics, attitudes/evaluations, behaviors, organizational characteristics, contextual characteristics, miscellaneous, intentions, dyad/group characteristics, HR practices, cognitions, and occupational characteristics), which branch to second-level nodes, third-level nodes, and so forth It presents what a variable represents rather than how it is used in the studies. An example of hierarchical classification used in the taxonomy developed by Bosco et al. (2015) is the variable transformative leadership located as a fifth-level node, where level one is the variable “behaviors,” level two is the variable “as an employee,” level three is the variable “leadership,” and level four is the variable “relations behavior.” The taxonomy offers the possibility to extract effect sizes at many different levels of generality by zooming in on each of the broad categories. Bosco et al.'s (2015) analyses were based on a database of 1,660 unique articles containing 25,891 variables, their associated effect sizes, and existing typologies in applied psychology research (Bosco et al., 2015). Thus, this study offers information that can be used to understand the relative importance of the effect sizes found in a particular study in relation to others in the same and other domains. We believe that this hierarchical taxonomy of variables, based on 147,328 correlations among the above-mentioned variables reported in the Journal of Applied Psychology and Personnel Psychology from 20 common research domains, represents a valuable approach to coaching outcomes classification and evaluation of the relative coaching effect on different levels of generality. The increased awareness regarding effect sizes across areas commonly researched in applied psychology allows us to anticipate a typical context-specific ES (e.g., for an attitude–behavior relation) and formulate predictors for observed coaching outcomes variables. As Wilkinson and the APA Task Force on Statistical Inference (Wilkinson, 1999, p. 599) noted, “We must stress again that reporting and interpreting effect sizes in the context of previously reported effects is essential to good research. It enables readers to evaluate the stability of results across samples, designs, and analyses.” Thus, a consistent classification of outcomes is crucial.

**Figure 1:**
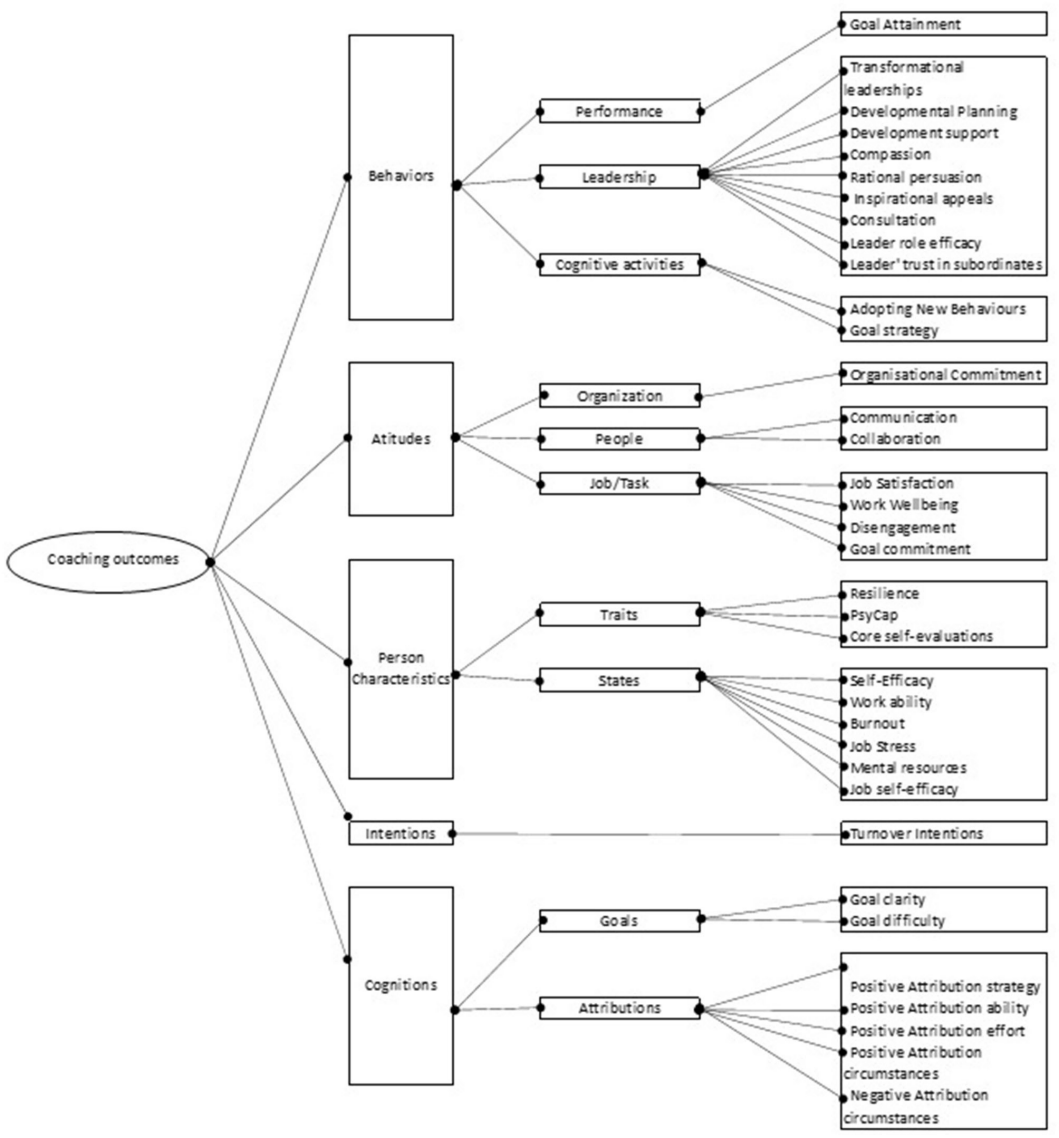
Executive coaching outcomes classification.

An overview of the coaching outcomes reported in the reviewed studies using the MetaBUS hierarchical structure is presented in Figure 1. As an example of hierarchical structure, the first level of coaching outcomes includes five general types: behaviors, attitudes, person characteristics, intentions, and cognitions. The second classification level includes sub-types such as leadership behaviors, organizational attitudes, and traits. The level of specificity further increases at the third classification level, which includes the individual outcomes variables reported in the reviewed studies classified according to the classification in Bosco et al.'s (2015) taxonomy. An example of a third-level outcomes variable is the goal attainment reported in four studies included in the current meta-analysis (Grant et al., 2009, 2010; Ballesteros-Sánchez et al., 2019; Zanchetta et al., 2020). The definitions of the types of outcomes included in the classification and the variables related to each type of outcome are listed in the Appendix.

### Hypotheses

The first study hypothesis flows from our classification model indicated in Figure 1. We expect to be able to show the effect size of executive coaching on overall outcomes. The previous meta-analyses found a moderate to large effect size for the overall coaching effectiveness. By including studies with rigorous research design, and a sample similar to the one used in the previous meta-analyses, we expect to find strong evidence of coaching effectiveness. Therefore:

*Hypothesis 1*. Executive coaching will demonstrate an overall positive significant effect size across all outcomes.

Based on the outcome classification proposed, we expected a meta-analysis of rigorous methodological design studies to be able to show the relative strength of executive coaching on different types of outcomes. Past research suggested that executive coaching is related to sustainable positive changes (Grant, 2012) and desirable behaviors (Ladegard and Gjerde, 2014; McGonagle et al., 2014; Nicolau and Constantin, 2021). Executive coaching was also associated with changes in attitudes, such as organizational commitment (Fontes and Dello Russo, 2021), and personality traits, such as core self-evaluations (McGonagle et al., 2014). However, attitudes and personality traits have been considered to persist despite attempts at change. A recent review on attitudes change found a small effect size (*d* = 0.22) for the attitude change based on interventions or messages delivered at a particular time (Albarracin and Shavitt, 2018), while interventions associated with marked changes in personality trait measures over time indicated a medium effect (*d* = 0.37) in a recent systematic review (Roberts et al., 2017). Previous meta-analyses indicated that the coachee behavioral change improvements were significantly larger than attitudinal changes (Sonesh et al., 2015), and the coaching outcomes represent the translation of learning through to performance benefits (Jones et al., 2016); therefore, we hypothesize:

*Hypothesis 2*: The relative strength of executive coaching on behavioral outcomes will be stronger compared to (a) attitude and (b) person characteristics outcomes.

Furthermore, executive coaching measurement usually relates to the managers' and organizations' bottom-line performance (Athanasopoulou and Dopson, 2018). In our classification, performance behaviors comprised goal attainment outcomes. Several studies have shown that coaching is associated with significant progress toward self-set goals (Grant et al., 2009; Grant, 2010; Ballesteros-Sánchez et al., 2019; Zanchetta et al., 2020). Moreover, when the coach guides managers through exploring their potential for development and optimal functioning, these managers usually define specific goals, explore internal and external resources, and find specific actions to achieve them (Schunk, 1995; Whitmore, 2002). Thus, behaviors include cognitive activities, such as goal strategy and adopting new behaviors, which also improve during coaching (Finn, 2007; Moen and Federici, 2012). The development of the alliance between the coach and coachee may also support behavioral modeling translated into the manager's leadership behaviors, such as openness to consultation and compassion (Kochanowski et al., 2010; McGonagle et al., 2020). However, executive coaching is a solution-focused goal-oriented process (Grant et al., 2009). Moreover, the hierarchical taxonomy developed by Bosco et al. (2015) based on effect sizes indicates that the performance behaviors (e.g., task performance) as an employee are better predicted than leadership behaviors (e.g., relations or change behaviors). Thus, we expect performance behaviors to be highly impacted in the coaching process. Therefore, we hypothesize:

*Hypothesis 3*: The relative strength of coaching on behavioral performance outcomes will be stronger compared to (a) behavioral leadership and (b) behavioral cognitive activities.

### Potential moderators

Several moderators of coaching effectiveness have been suggested, such as coach background, coaching model, type of coach (external or internal), and the number of coaching sessions. For most of these potential moderators, the lack of data reported in the reviewed studies prevented us from including them in the meta-analysis. For example, regarding coaching methods, we evaluated the methods reported in the included studies, such as positive psychology coaching, Goal-Reality-Options-Will (GROW), and cognitive-behavioral developmental coaching. Several of these methods' components, such as setting the coaching goal, defining strategies, designing actions for achieving the goal, and identifying resources or strengths, and overlap. Therefore, we concluded that there are no clear distinctions among them. Two related aspects of coaching could be included, i.e., the number of coaching sessions and the overall length of the coaching program. Research examining these possible moderators is limited and sometimes contradictory. For example, some meta-analyses suggested that the number of coaching sessions or the overall longevity of coaching interventions did not significantly impact the executive coaching outcomes (Theeboom et al., 2014; Jones et al., 2016), while others showed a significant moderating effect of the number of coaching sessions (Sonesh et al., 2015). Authors have suggested that even a one-time coaching session is effective (Bright and Crockett, 2012). It is possible that executive coaching may have an immediate effect by directing managers' resources and focusing on clarifying the actions that are needed to increase performance (Moen and Skaalvik, 2009; Bright and Crockett, 2012; Peláez Zuberbuhler et al., 2020). In their meta-analysis, Sonesh et al. (2015) suggested that the complexity and difficulty of the coaching goals may impact the number of coaching sessions. As participants acquired new behaviors and competencies, they inevitably developed feelings of self-efficacy, goal-focused orientation, and positive affect, which, once influenced, continue to increase over time, generating a “prophylactic effect” (Finn, 2007; Grant, 2010). Given these considerations, we did not expect unambiguous effects of the number of sessions and length of the coaching program on the coaching outcomes, and we did not formulate a hypothesis regarding these moderation analyses.

## Method

### Literature search and inclusion criteria

**Figure 2:**
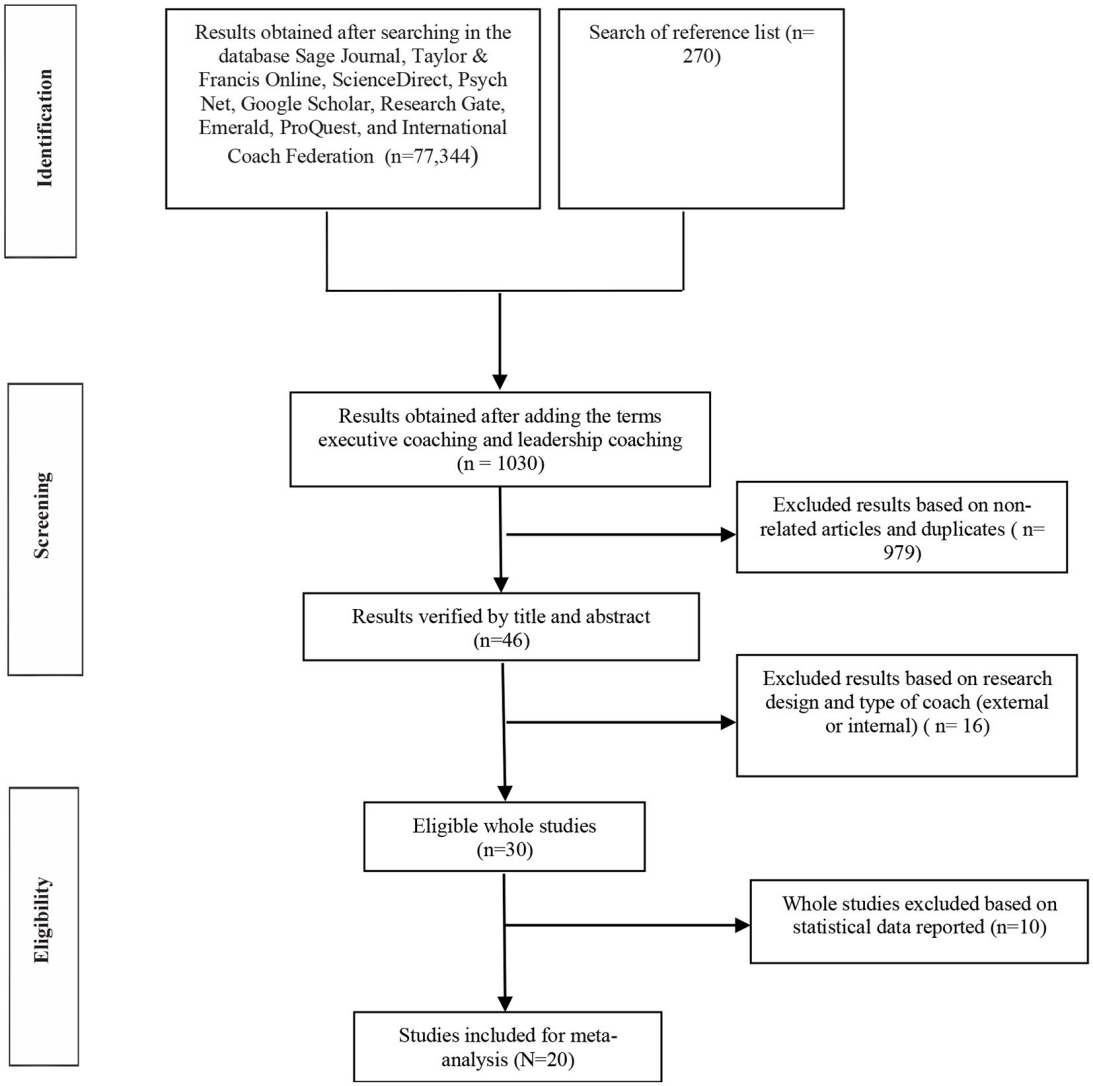
PRISMA flowchart.

In this study, we focus on executive coaching as a supportive relationship provided by an external professional coach and a manager with authority and responsibility in an organizational context, excluding all other coaching or developmental practices (e.g., life coaching, internal/managerial coaching, and mentoring). To identify relevant published and unpublished studies (including doctoral theses), science databases were searched (SAGE Journals Online, Taylor and Francis, Science-Direct, PsycINFO, Google Scholar, Emerald, Research Gate, ProQuest, and ICF Research Portal) using the following keywords: “executive coaching,” “leadership coaching,” “managerial coaching,” “coaching” in combination with “organizational performance.” We additionally manually reviewed the reference lists of all publications identified in the database search and the studies cited in the previous meta-analyses (i.e., De Meuse et al., 2009; Theeboom et al., 2014; Sonesh et al., 2015; Jones et al., 2016; Burt and Talati, 2017) and included the relevant studies. The Preferred Reporting Items guided this extensive literature search for Systematic Reviews and Meta-Analyses (PRISMA; Liberati et al., 2009, see Figure 2).

We assessed the studies for inclusion based on the following criteria: (a) the executive coaching intervention matched Kilburg's (1996: p. 142) definition; (b) an external coach led the executive coaching intervention in a one-to-one session in an organizational setting; (c) the published or unpublished study was in English; (d) the study empirically investigated the relationship between the intervention and the executive coaching outcomes for the client; (e) the study used a between-subject design with a pre-post-test and control group and reported sufficient statistical data such as group sample sizes, means and standard deviation for each group.

The main reason for not including studies that used internal coaches was to control for effects that may contaminate the overall result of coaching effectiveness. Jones et al. (2016) suggested that the effects of coaching by internal coaches had a stronger effect compared to external coaches. Moreover, the research on mentoring has shown that a mentee's behavior can be affected during the session when the mentor has formal authority over the mentee (Mullen, 1994). Thus, we decided to include only studies that used external coaches.

The current meta-analysis did not include a study if the data needed to establish effect sizes were unavailable.

The first two authors independently selected potentially eligible articles. The final set of records was selected by reading the full texts of all articles that passed an initial screening of titles and abstracts. There was a 95.3% initial interrater agreement for screening full texts.

The disagreements about screening and selection were resolved by consensus.

The selection process yielded a total of 20 studies that met the selection criteria and were included in the meta-analysis (see Figure 2 and Table 1). All studies included in the final analysis are indicated with an asterisk in our list of references.

**Table 1:** Inclusion criteria, outcomes, and moderators in previous meta-analyses and the current study.

**Meta-analysis**	**Nr of studies included**	**Inclusion criteria**	**Design of the included studies**	**Outcomes**	**Moderators**
Current meta-analysis	20	- Executive coaching matched Kilburg's (1996) definition	RCT	- Behaviors Cognitive activities Leadership Performance	- Number of coaching sessions - Length of coaching program (weeks)
		- External coach		- Attitudes Organization People Job/Task	
		- Randomized control trial		- Person characteristic States Traits	
		- Sufficient statistics reported			
Burt and Talati (2017)	11	- Coaching method design equivalent to Grant's (2003) definition	RCT	- Attitudes	-Participant gende
		- External coach		- Coping	
		- Randomized control trial		- Self-regulation	
		- Outcomes reported		- Well-being	
		- Sufficient statistics reported			
Jones et al. (2016)	17	- Workplace coaching	RCT	- Affective outcomes	- Research design
		- Within- and between-subjects designs	QEF	- Skill-based outcomes	- Multisource feedback
		- Report sample sizes	WSD	- Individual level outcomes	- Format of coaching
		-Reported statistics			- Type of coach (internal or external)
		- Coaching outcome measured at individual level			- Number of coaching sessions
Sonesh et al. (2015)	24	- Leadership, business, or executive coaching	RCT	- Relationship outcomes	- Sample type
		- Empirical studies	QEF	Generic coach–coachee relationship	- Design type
			WSD	Working alliance	- Coach background
				- Coachee outcomes	- Coach expertise
				Goal attainment	- Number of coaching sessions
				Behavioral change	
				Work-related attitude change	
				Personal attitude change	
				Improved relations with others	
				Overall satisfaction with coaching	
				Cognitive change	
				Task performance	
				- Organization outcomes	
Theeboom et al. (2014)	18	- External professional trained coach	RCT	- Performance/skills	- Study design
		- Studies conducted in a work or educational context	QEF	- Work attitude	- Number of coaching sessions
		- Coaches belonged to a non-clinical population	WSD	- Coping	
		- No influence of other interventions		- Self-regulation	
		- Enough statistical information		- Wellbeing	
		- Included quantitative data			
De Meuse et al. (2009)	12	- Executive coaching	QEF	- Reactions to coaching	- Type of coaching provided (suggested)
		- External coach	WSD	- Coaching effectiveness at individual level	- Content of the coaching engagement (suggested)
		- Pre- and post-coaching ratings		- Coaching impact at organizational level	- Content of the coaching engagement (suggested)
		- Statistics reported in the article			

RCT, randomized control trial; QEF, quasi-experimental field study in which participants were non randomly allocated to experimental and control groups; WSD, within-subjects design without control group, which includes both pre- and post-intervention measures.

### Research model

The statistical model used to conduct the meta-analysis was a random effects model, which permits that the true effect sizes vary from study to study based on both the independent variable and the variability and differences in the research sample (Hedges and Cooper, 1994; Borenstein et al., 2009). The classical Cochran Q statistic and the *I*^2^ statistic, as proposed by Higgins and Thompson (2002), were calculated to test for heterogeneity between studies. The Q statistics provide a test of significance for between-study heterogeneity, while the value of *I*^2^ represents the proportion of between-study variance in effect sizes to between-study heterogeneity (Borenstein et al., 2009). When the value for *I*^2^ is close to or above 50%, moderating variables are considered to be present (Higgins and Thompson, 2002). Moderator analysis was conducted using sub-group analysis when the moderators were categorical and meta-regression when the moderators were continuous.

### Outcome categories and moderators

The first two authors independently codded the outcomes. The disagreements about outcomes coding were resolved by consensus. The categorization of outcomes was conducted in two steps. In the first step, we assigned the studies to one of the following general executive coaching outcomes categories: behaviors, attitudes, person characteristics, cognitions, and intentions. In the second step, we distinguished 10 sub-types categories based on the taxonomy developed by Bosco et al. (2015).

We eliminated two general types of outcomes that could not be tested because of the limited number of studies that included them: cognition and intentions. Several studies selected for the meta-analysis included a broad range of outcomes within the same outcome category (e.g., measures of leading and effectiveness both fall within the behavior category). For those studies, we examined the individual effect sizes for each category, and an overall effect size per study was included for each outcome category. All reported outcomes in the primary studies are based on self-reports and not on observations of others (e.g., subordinates and peers), test results, or measures of job performance because other measures were either not reported or reported only in a few studies.

In addition to the meta-analysis of the executive coaching outcomes, we investigated whether the length of the coaching program and the number of sessions moderate the effectiveness of executive coaching.

### Effect sizes, methodology, and database

Similar to the previous meta-analyses, the overall weighted effect size was calculated using the Hedges and Olkin (1984) approach. The post-pre effect size from the control group was subtracted from the post-pre effect size for the treatment group. To standardize the mean difference between the pre- and post-measures of coaching outcomes, the effect sizes of the original studies were transformed to a variation of Cohen's *d*, the Hedges' *g* effect size. This conservative approach allows for statistical correction of biases due to small sample sizes, measurement error, and range restriction to avoid overestimates (Hedges, 1981; Hedges et al., 2009). It provides a conservative estimate of the confidence interval used for calculating the statistical significance of effect sizes (Johnson et al., 1995).

We used Biostat's Comprehensive Meta-Analysis program (CMA) version 2 (Borenstein et al., 2005). This application is based on the approach to meta-analysis proposed by Hedges and Olkin (1984). CMA offers the advantages of handling multiple data entry formats, the detection of between-study heterogeneity, and an intuitive approach to sensitivity analysis (Borenstein, 2005).

We described our sampling plan, all data exclusions (if any), all manipulations, and all measures in the study. All data, analysis codes, and research materials are available at: https://osf.io/pz2a6/?view_only=1d4a77d2121b472f86fbd8def20f2def. The study design, hypotheses, and analysis plan of the reported work were not preregistered.

## Results

### Aggregated effect sizes across all studies and outcomes

Table 2 contains the weighted effect sizes (aggregated across outcomes) per study. We found an overall moderate weighted effect size for all outcomes, reflecting an advantage of coaching over control groups [Hedges' *g* = 0.43, 95% CI, 0.35–0.50, *p* < 0.001 [95% PI (−0.05 to 0.92)]. Hypothesis 1 was clearly supported. The results show that coaching has a significant positive effect on the overall coachee outcomes. The results also indicate that heterogeneity was statistically significant and medium in magnitude (*Q* = 100.64; *p* < 0.001; *I*^2^ = 61.24). Therefore, we continued this analysis by exploring various moderators for the positive effect of executive coaching.

**Table 2:** Weighted effect sizes aggregated over outcomes per study.

	***N***	**Hedges** ***g***	**Standard error**	**95% CI**		***Z*** **-value**	***p***
				**LL CI**	**UL CI**		
Ballesteros-Sánchez et al. (2019)	30	0.44	0.15	0.15	0.73	3.01	0.003
Bright and Crockett (2012)	127	0.24	0.06	−0.18	0.78	1.21	0.226
Evers et al. (2006)	60	0.26	0.10	0.05	0.46	2.44	0.014
Finn (2007)	17	0.98	0.29	0.41	1.56	3.36	0.001
Fontes and Dello Russo (2021)	53	0.44	0.13	0.19	0.70	3.38	0.001
Grant et al. (2009)	41	0.61	0.20	0.21	1.01	3.00	0.003
Grant et al. (2010).	44	0.92	0.21	0.52	1.32	4.47	< 0.001
Kochanowski et al. (2010)	93	0.12	0.13	−0.13	0.37	0.95	0.340
Ladegard and Gjerde (2014)	30	1.50	0.34	0.83	2.18	4.37	0.000
MacKie (2014)	31	0.38	0.36	−0.32	1.07	1.06	0.287
McGonagle et al. (2014)	48	0.53	0.15	0.23	0.83	3.44	0.001
McGonagle et al. (2020)	50	0.38	0.11	0.17	0.59	3.58	< 0.001
Moen and Federici (2012)	19	1.07	0.24	0.60	1.54	4.46	< 0.001
Moen and Skaalvik (2009)	108	0.40	0.15	0.10	0.70	2.58	0.010
O'Connor and Cavanagh (2013)	102	0.30	0.25	−0.19	0.79	1.21	0.227
Richardson (2010)	18	0.62	0.35	−0.05	1.30	1.81	0.070
Williams and Lowman (2018)	32	0.57	0.17	0.23	0.92	3.25	0.001
Smither et al. (2003)	1,229	0.35	0.12	−0.67	0.69	0.03	0.973
Zanchetta et al. (2020)	70	0.86	0.17	0.52	1.20	4.92	< 0.001
Peláez Zuberbuhler et al. (2020)	38	0.73	0.19	0.35	1.11	3.77	< 0.001
**Random effects model**		**0.43**	**0.04**	**0.35**	**0.51**	**11/05**	**< 0.001**

*N*, analyzed sample size; LLCI, lower limit for the 95% confidence interval for g; ULCI, upper limit for the 95% confidence interval for g. The bold values indicate the aggregated results across outcomes.

### Effect sizes per general type, sub-type, and specific outcome

Table 3 presents the effect sizes for coaching interventions on the three general types of outcomes: behaviors, attitudes, and person characteristics. The effect of coaching was significant and positive for all three categories. The effect sizes of coaching on behaviors, attitudes, and person characteristics were all positive (behaviors: Hedges' *g* = 0.73, 95% CI, 0.41–1.05, *p* < 0.001; attitudes: Hedges' *g* = 0.34, 95% CI, 0.24–0.44, *p* < 0.001; person characteristics: Hedges' *g* = 0.51, 95% CI, 0.39–0.64, *p* < 0.001). We compared the effects between these three categories. The results indicated a significant difference between attitude and behavioral outcomes categories (*Q* = 5.167, *p* < 0.05) and attitudes and person characteristics categories (*Q* = 4.205, *p* < 0.05). These results supported Hypothesis 2a regarding the relative effect of coaching on behavioral outcomes compared with attitudes and person characteristics outcomes. We also observed low heterogeneity in the results for attitudes and person characteristics (non-significant Q values and low *I*^2^). However, we found significant heterogeneity for behavior (*Q* = 60.48, *p* < 0.001; *I*^2^ = 81.81), which suggested possible moderators.

**Table 3:** Effect sizes for general types outcomes: behaviors, attitudes, and person characteristics.

**Outcome**	**Samples**	***N***	**Hedges** ***g***	**Standard error**	**95% CI**		***Z*** **-value**	***p***	***Q***	*I* ^2^
					**LL CI**	**UL CI**				
Behavior	12	565	0.73	0.16	0.42	1.07	4.53	< 0.001	58.90^***^	81.32
Attitudes	12	1,793	0.34	0.05	0.24	0.44	6.61	< 0.001	10.79	0.00
Person characteristics	16	830	0.52	0.06	0.39	0.65	7.88	< 0.001	24.64	39.12
**Between types**										
Behavior vs. attitudes									5.16^**^	
Behavior vs. person characteristics									1.53	
Attitudes vs. person characteristics									4.20^**^	

*N*, total sample size in k studies; LLCI, lower limit for the 95% confidence interval for g; ULCI, upper limit for the 95% confidence interval for g; Q, Cochran Q statistic.

I^2^, the proportion of total variation in the estimates of treatment effect that is due to heterogeneity between studies.

*** Indicates that between-study heterogeneity is significant (*p* < 0.001).

** Indicates that between-study heterogeneity is significant (*p* < 0.05).

The results for the relative effects of coaching on behavioral outcome sub-categories yielded a larger effect size for the cognitive activities outcomes than for performance outcomes and a significant difference between cognitive activities and leadership activities (*Q* = 10.388, *p* < 0.01). Hypothesis 2a was not supported. Table 4 presents the effect sizes of the coaching intervention on sub-categories.

**Table 4:** Effect sizes for sub-types outcomes.

**Outcome**	**Samples**	***N***	**Hedges** ***g***	**Standard error**	**95% CI**		***Z*** **-value**	***p***	***Q***	*I* ^2^
					**LL CI**	**UL CI**				
**Behavior**										
Cognitive activities	3	144	1.28	0.17	0.94	1.62	7.36	< 0.001	0.35	0.00
Leadership	7	283	0.44	0.19	0.07	0.82	2.33	< 0.010	19.59^**^	69.38
Performance	4	185	1.11	0.28	0.55	1.68	3.86	< 0.001	11.47^**^	73.85
**Between sub-types**										
Cognitive vs. leadership									10.38^**^	
Cognitive vs. performance									0.23	
Performance vs. leadership									3.74	
**Attitudes**										
Organization	1	44	0.47	0.30	−0.11	1.06	1.58	0.112	0.00	0.00
People	7	1,550	0.30	0.13	0.04	0.57	2.27	< 0.050	8.33	27.97
Job/task	7	382	0.39	0.06	0.26	0.52	5.94	< 0.001	3.65	0.00
**Between sub-types**										
People vs. job/task									0.31	
**Person characteristics**										
States	13		0.50	0.07	0.36	0.64	7.04	< 0.001	18.38	34.72
Traits	7		0.61	0.13	0.34	0.88	4.44	< 0.001	10.92	45.06
**Between sub-types**										
States vs. traits									0.47	

*N*, total sample size in k studies; LLCI, lower limit for the 95% confidence interval for g; ULCI, upper limit for the 95% confidence interval for g; Q, Cochran Q statistic.

I^2^, the proportion of total variation in the estimates of treatment effect that is due to heterogeneity between studies.

** Indicates that between-study heterogeneity is significant (*p* < 0.05).

We computed a series of analyses for some specific outcomes (see Table 5). Although most studies involved the measurement of multiple outcomes, only a few of them were included in a sufficient number of studies to allow us to do meaningful meta-analytic calculations. Thus, we found significant and positive effects on self-efficacy (Hedges' *g* = 0.31, *p* < 0.05), goal attainment (Hedges' *g* = 0.32, *p* < 0.001), psychological capital (Hedges' *g* = 0.83, *p* < 0.001), and resilience (Hedges' *g* = 0.57, *p* < 0.001). The aggregated effect size for job satisfaction was positive but not significant (Hedges' *g* = 0.36, *p* = 0.17).

**Table 5:** Effect sizes for specific outcomes.

**Outcome**	**Samples**	***N***	**Hedges** ***g***	**Standard error**	**95% CI**		***Z*** **-value**	***p***	***Q***	*I* ^2^
					**LL CI**	**UL CI**				
Self-efficacy	5	254	0.31	0.13	0.04	0.58	2.25	0.02	5.78	30.88
Goal attainment	3	155	1.32	0.35	0.63	2.00	3.77	< 0.001	7.46^*^	73.19
Job satisfaction	3	154	0.36	0.27	−0.17	0.91	1.31	0.17	5.86	65.92
PsyCap	3	144	0.83	0.17	0.49	1.17	4.80	< 0.001	1.84	0.00
Resilience	3	133	0.57	0.17	0.23	0.91	3.31	< 0.001	0.34	0.00

*N*, total sample size in k studies; LLCI, lower limit for the 95% confidence interval for g; ULCI, upper limit for the 95% confidence interval for g; Q, Cochran Q statistic.

I^2^, the proportion of total variation in the estimates of treatment effect that is due to heterogeneity between studies.

* Indicates that between-study heterogeneity significant (*p* < 0.05).

### Moderator analysis

We tested the moderating role of the length of the coaching program and the number of sessions for the three outcome categories (behavior, attitudes, and person characteristics). The results are displayed in Table 6. A series of meta-regressions showed that the length of the coaching program was a significant moderator only for the attitudes category (coefficient = 0.01, se = 0.008, *p* = 0.03). The longer the program was, the greater the effect size. The number of sessions was not a significant moderator for any outcome category.

**Table 6:** Results for the moderators.

**Outcome**	**Samples**	**Coefficient**	**Standard error**	**95% CI**		***z***	***p***
				**LL CI**	**UL CI**		
**Behavior**							
Number of sessions	12	0.11	0.09	−0.06	0.29	1.25	0.21
Length of program	12	0.03	0.01	−0.001	0.06	1.96	0.05
**Attitudes**							
Number of sessions	12	0.004	0.03	−0.05	0.06	0.13	0.89
Length of program	10	0.01	0.008	0.001	0.03	2.11	0.03
**Person characteristics**							
Number of sessions	15	0.001	0.02	−0.05	0.05	0.03	0.97
Length of program	14	−0.001	0.01	−0.02	0.01	−0.08	0.93

LLCI, lower limit for the 95% confidence interval for the coefficient; ULCI, upper limit for the 95% confidence interval for coefficient.

### Publication bias

Publication bias was assessed via visual inspection of funnel plots and the Egger bias test (Egger et al., 1997). The Egger test was significant (*p* < 0.001). Based on this result, we conducted further sensitivity analyses. Firstly, using the trim and fill method, we found that by adding 29 new effect sizes in the meta-analysis, the aggregated effect size would decrease from 0.43 to 0.27. Even with this decrease, the aggregated effect size would remain similar. Secondly, by using Orwin's fail-safe N method (Orwin, 1983), we found that to find a trivial effect size (<0.10), the addition of 332 studies with an effect size lower than 0.10 would be necessary. Concluding, we found no publication bias in this study.

## Discussion

The purpose of the current meta-analysis was to evaluate coaching effectiveness by investigating its effect on the overall outcomes studied and its relative strength on different coaching outcome types by systematically reviewing the existing RCT studies on coaching effectiveness at work. Additionally, the study examined whether the number of coaching sessions and overall length of the coaching program moderated its effectiveness. To evaluate the executive coaching intervention and multiple types of outcomes, we proposed a classification based on previously used taxonomy in applied psychology. Hypothesis 1 was confirmed. We found strong evidence for a significant moderate effect of executive coaching on overall coaching outcomes dimensions reported in the reviewed studies. We found support for Hypothesis 2a and Hypothesis 2b. The results indicated that the relative strength of coaching was higher on behavioral than on attitude and person characteristics outcomes, with a significant difference between behavior and attitude outcomes and between attitude and person characteristics outcomes. Hypotheses 3a and 3b were not confirmed. The analyses indicated a large effect size for performance behaviors and behavioral-cognitive activities and a moderate effect size for leadership behaviors, with a significant difference only between behavioral cognitive activities and leadership behavior. The results show no moderating effects of the number of coaching sessions and the length of the coaching program, except for a positive relationship between the coaching program's length and coaching effectiveness for the attitude outcome. In summary, our meta-analytic findings indicate that executive coaching is an effective tool resulting mainly in changes in individuals' behaviors, especially in cognitive behaviors, with no conditional effect from the number of sessions or the length of the coaching program.

### Contribution to theory and future research

Our results are consistent with previous findings in the executive coaching literature about the overall effect size and the varying effect sizes for different outcomes (Theeboom et al., 2014; Jones et al., 2016; Burt and Talati, 2017). We found clear indications that the relative strength of coaching was higher on behavioral outcomes than on other outcomes categories. Considering the stability of attitudes and person characteristics, the lower effect sizes reported in our study can be expected. However, our findings on attitudes are likely to throw light on the contextual factors that contribute to coaching effectiveness. Improvements in work-related attitudes, such as job satisfaction and wellbeing, suggest that coaching may facilitate a better alignment between the individual and the organization, ultimately creating a positive impact on coachees' organization-related attitudes and the perception of coaching as an aid from the organization for individual development (Fontes and Dello Russo, 2021). The executives may experience positive work-related attitudes due to the perceived support provided by the organization that sponsors them with coaching (McGonagle et al., 2020). Managers who receive executive coaching may develop a stronger sense of control over their outcomes and workplace experience, resulting in an improved attitude and enhanced personal characteristics.

We suggest that future research continues to assess the impact of the organizational context on executive coaching outcomes. It would be interesting to explore if a higher alignment between change initiatives at the organizational level and managers' goals or increased support and resources from the supervisor would positively impact coaching outcomes.

We found confirmation that cognitive activities, such as openness to new behavior and goal strategy, represent the most impacted behavioral aspect. This is important because behavioral change is the most common objective of coaching engagements (Sonesh et al., 2015). Our findings suggest that although not the primary focus of executive coaching, cognitive outcomes, such as developing planning and goal strategy, are essential in promoting behavioral change. The coach's feedback and support, and the focus on planning, can lead the coachee to develop an action plan, find personal resources, and identify strategies to overcome possible obstacles (Fontes and Dello Russo, 2021). The large effect size (*g* = 1.28) for cognitive activities differs considerably from recent meta-analyses reported (*g* = 0.22; Sonesh et al., 2015). However, these results should be interpreted cautiously, given the small number of studies included in the analysis (*k* = 3).

While our meta-analysis provides an insight into the relative effects of executive coaching on the different types of behavior outcomes, we were unable to explore specific behavior relationship constructs (e.g., adopting new behaviors, leader trust in subordinates, rational persuasion, and inspirational appeal) or task-oriented ones (e.g., goal types) due to a lack of primary studies. The coaching field would benefit from defining outcomes by other considerations, essential for the behavioral outcome distinction given the variability in its effect sizes between studies. Moreover, future research may investigate how the coaching and control groups changed in different behavioral outcomes over time using multiple post-measures to establish whether executive coaching facilitates consistent change.

Our meta-analysis revealed significant and positive effects of executive coaching on specific outcomes such as goal attainment, self-efficacy, psychological capital, and resilience. Several mechanisms may be responsible for these improvements. Firstly, goalsetting at the starting phase of the coaching process affects goal attainment through direction, energy, persistence, and improved task strategies (Locke and Latham, 2002). Secondly, executive coaching may encompass psychosocial-support dimensions such as self-confidence or emotional support for executives' role distress (Nicolau and Constantin, 2021). It allows one to reflect on attempts and errors, gain awareness of personal resources and strengths (Rank and Gray, 2017), and strive for mastery experiences, leading to improved self-efficacy. By focusing on strengths and positive episodes, the reflection would increase confidence in development and change, the ability to identify options, optimism about the change, and resilience when confronted with obstacles (Fontes and Dello Russo, 2021), thus improving psychological capital. It boasts a solution-focused reflection and strategy with a significant impact on increasing resources and a sense of control through planning, which has the potential to reduce stress and enhance resilience (Grant et al., 2009; Nicolau and Constantin, 2021).

Nevertheless, executive coaching may exhibit the strongest relationship with these specific outcomes because they are assessed holistically, especially when assessing goal attainment (Graßmann et al., 2020). For example, goal achievements may depend on the manager's tasks, team dynamics, and organizational support. Considering that organizations sponsored the executives with coaching for personal development in the reviewed studies, we might assume that the managers had perceived high contextual support, thus remaining committed and engaged to their goals (Fontes and Dello Russo, 2021). Future research should investigate the impact of perceived low organizational support, destructive leadership, or disruptive team dynamics on coaching outcomes.

This study did not focus on the effectiveness of specific elements coaches involve in their coaching engagement. Past research suggested that coaching is a complex dynamic change process rather than a linear input-output mechanism (Erdös and Ramseyer, 2021; Wasylyshyn, 2022; de Haan and Nilsson, 2023) where relational coaching processes significantly impact the outcomes. Future research may want to investigate some coaching processes that lead to change, such as the goal type, the coachee's engagement during sessions, or the perceived coach's support impact on executive coaching effectiveness.

Moderator analyses resulted in only one significant moderator, the length of the coaching program, and only for the attitudes dimension. Although the length of the coaching programs may heighten the opportunity to identify resources and strategies for goal attainment, it is unclear what the moderating effect means, as we did not find differences in the number of coaching sessions. A possible explanation could be related to the fact that attitudes and beliefs that are consistent with other values are likely to persist, and defensive cognitive processes can be involved to protect the attitudes and beliefs, especially when motivation is low (Hart et al., 2009). This means managers who receive coaching might need more time to change their attitudes regardless of the number of sessions. Future research should investigate how the length of the coaching program may alter or enhance the coaching outcomes. Furthermore, researchers should provide more details related to the goal type, motivation, and organizational context to allow future analyses of other possible moderators.

The effect sizes observed across different types of outcomes allow for identifying the research area that lags. As described earlier, we eliminated from the analyses two types of outcomes, intentions and cognitions, and subtypes of outcomes, such as goals and attitudes, because of the limited number of studies that included them. We propose that for coaching research to continue to advance, these are areas where progress is not being made and thus constitute important directions for future studies.

### Contribution to the coaching practice

We found a robust relationship between executive coaching and all the outcome dimensions investigated, suggesting that executive coaching is an effective developmental tool from which all organizations may benefit. As an implication for practice, we suggest that organizations use executive coaching as a valuable tool for managers in any context, especially during organizational change, given both business and human costs (Grant et al., 2009). Our study points to behavior cognitive activities as the main outcome categories impacted by coaching. Thus, organizations may consider using coaching for managers involved in planning and strategizing processes, such as product and project managers. Moreover, designing coaching interventions that help managers identify goal strategies and support developmental planning in their roles and relationships at work could have significant consequences at the organizational level (O'Connor and Cavanagh, 2013).

Considering the possible change in attitudes and person characteristics, we suggest that the strength of the coaching on these dimensions could be stronger if coaches take a more systemic approach and facilitate aligning the manager's goals with the organization's strategy to enhance their positive attitudes toward the organization and, thus, motivation and engagement in goal achievement.

We found confirmation that the number of coaching sessions does not impact the coaching effectiveness. This suggests that the coachees may adjust the number of sessions based on their needs and the complexity of the goals. This coregulation phenomenon was supported in psychotherapy by research that found that the patients showed similar gains regardless of the duration of the treatment (Stiles et al., 2015). Therefore, we suggest that allowing the coachees to schedule their own appointments rather than scheduling regular (e.g., weekly or bi-weekly) appointments might significantly reduce “no-shows” or session cancellations without effects on coaching effectiveness.

### Limitations

Although many of the methodological limitations of previous meta-analytic studies were addressed, some notable ones remained. Firstly, the coaching interventions, measures, and outcomes across the included studies varied considerably, which limited the number of direct comparisons that could be made for a meta-analytic synthesis. Some of the findings are based on a relatively small number of studies (k = 3), and such estimates are likely to be less stable (Borenstein et al., 2009). Furthermore, we could not analyze the relationship with executive coaching for some outcome dimensions, such as cognitions and intentions, or potential moderators, such as coaching background and coaching method, because of missing studies or data in the reviewed studies. Although there was no evidence for publication bias and the inclusion of the 20 studies was above the minimum for meta-analysis (Sterne et al., 2008), further research is still required to substantiate these results and to explore the uncovered outcomes dimensions and possible moderators. Secondly, even though we provide insights into the impact of executive coaching on behaviors, attitudes, and person characteristic dimensions, we were unable to analyze specific outcomes (e.g., leading, engagement, job stress, and turnover intentions) or specific achievement goal frameworks due to a lack of primary studies. Thirdly, we could not measure the possible impact of executive coaching on other people within an organization due to the focus on individual outcomes of coaching in the studies included in our analysis (Grant et al., 2009). Similarly, we could not include the possible impact of context factors in facilitating or thwarting the coaching outcomes (Fontes and Dello Russo, 2021). Fourthly, the authors of the reviewed studies have investigated the coaching outcomes using a variety of measurements that could have influenced the results and may have depended on other variables (Graßmann et al., 2020).

## Conclusion

The current meta-analysis strongly contributes to providing evidence that executive coaching is an effective intervention in organizations, especially for behavioral change. While the literature shows that the recipients of executive coaching typically are leaders (De Meuse et al., 2009; MacKie, 2014; de Haan and Nilsson, 2023), this meta-analysis has brought significant support for the effectiveness of coaching that organizations may consider using for other groups of employees, such as production employees, to support their personal development, mainly due to its impact on behavioral cognitive activities and performance behaviors such as goal strategy and goal attainment.

Despite some limitations, this meta-analysis has made important contributions to our understanding of coaching effectiveness. Firstly, it proposes a way to structure the large variety of outcomes using a systemic approach for outcomes classification based on existing typologies in applied psychology research (Bosco et al., 2015). This meta-analysis provides a more inclusive and robust hierarchic classification framework for coaching outcomes and points to areas less explored by coaching research. It highlights future research directions, such as intentions, goals, cognitions, and attributions. Secondly, it investigates the relative effect of executive coaching on different types of coaching outcomes reported in RCT studies. Unlike the previous meta-analyses, it includes only the studies on executive coaching in organizational settings with rigorous research designs that allow controlling for a range of threats to internal and external validity (Grant et al., 2009; Grant, 2012) and substantially reduce same-source biases, Hawthorne effects, and other false positives. Thirdly, our study offers evidence regarding the distribution of effect sizes across different coaching outcomes and compares the strengths of coaching on different types of outcomes to assess what is most impacted. Fourthly, it explores possible conditions for the relative effect of coaching on different types of outcomes, providing evidence for the importance of these conditions for coaching effectiveness.

Overall, our study supports the further development of coaching research and practice that can be used to identify the underlying mechanisms and processes by which coaching interventions are successful.

## Data availability statement

The datasets presented in this study can be found in online repositories. The names of the repository/repositories and accession number(s) can be found in the article/Supplementary material.

## Author contributions

AN and TC contributed to the conception and design of the study. AN organized the database and wrote the first draft of the manuscript. OC and AN performed the statistical analysis. AN, OC, and AK wrote sections of the manuscript. All authors contributed to the manuscript revision, read, and approved the submitted version.
