# Supplementary material for: The effects of executive coaching on behaviors, attitudes, and personal characteristics: a meta-analysis of randomized control trial studies
Source: Front Psychol. 2023 Jun 2;14:1089797. doi: 10.3389/fpsyg.2023.1089797 (PMC10272735; doi:10.3389/fpsyg.2023.1089797)
Supplement: Supplementary file 1 [file Data_Sheet_1.docx]

**Appendix**

*Definitions and variables related to types of outcomes*

| Types of outcomes | | Definition* | Example of variables related to the type** |
| --- | --- | --- | --- |
| **General types** | **Sub-types** |  |  |
| - Behaviors |  | A person's activities in response to external or internal stimuli, including objectively observable activities, introspectively observable activities, and nonconscious processes | Performance, Leadership, Interaction behavior, Counterproductive, Movement, Absenteism, Cognitive activities, Participative behaviors |
|  | - Performance | Any activity or collection of responses that leads to a result or has an effect on the environment | Effort, Decision making, Problem-solving, Job knowledge, Initiative, ***Goal Attainment,*** Productivity |
|  | - Leadership | The processes involved in leading others, including organizing, directing, coordinating, and motivating their efforts toward achieving certain group or organizational goal | ***Transformational leadership***, Mentoring, Empowerment, ***Developmental Planning***, Servant leadership, ***Development support***, ***Compassion,***  ***Rational persuasion, Inspirational appeals, Consultation, Leader role efficacy, Leader's trust in subordinates,*** Leader emergence, Nonleadership |
|  | - Cognitive activities | The behaviors involved in performing the tasks associated with learning, memory, understanding, awareness, reasoning, and judgment | Goal setting, ***Goal Strategy,*** Active maintenance approach, ***Adopting new behaviors,*** Group strategy, Problem-solving coping, Emotional control strategy, Organizing, Product planning, Account planning, Metacognitive activities |
| - Attitudes |  | A relatively enduring and general evaluation of an object, person, group, issue, or concept on a dimension ranging from negative to positive. | ***Organizational, Job/Task, People,*** Career, Group/processes, Training, Communication, Change |
|  | - Organization | A relatively enduring and general evaluation of an organization on a dimension ranging from negative to positive | Loyalty, Support, Trust, Engagement, ***Commitment***, Dedication, Attachment |
|  | - People | A relatively enduring and general evaluation of a person or group on a dimension ranging from negative to positive | Relationship quality, Cooperation, Interpersonal treatment, Cohesiveness, ***Collaboration, Communication***, Bonding, Social capital, Destructive relationships |
|  | - Job/Task | A relatively enduring and general evaluation of a job or task on a dimension ranging from negative to positive | Positive job affect, ***Job satisfaction, Work wellbeing,*** Need satisfaction, Negative job affect, ***Disengagement***, Feedback, ***Goal commitment*** |
| - Person Characteristics | | A particular feature or quality of a person, especially any of the enduring qualities or traits that define an individual’s nature or personality in relation to others | Psychological, as an employee, Objective characteristics |
|  | - Traits | An enduring personality characteristic that describes or determines an individual’s behavior across a range of situations | Personality, Trait orientation, Traits self-efficacy, Engagement, Locus of control, ***Core self-evaluation, Psychological Capital, Resilience,*** Self-monitoring |
|  | - States | The condition or status of a person at a particular time that is characterized by the relative stability of its basic components or elements | Confidence, Happiness, Enthusiasm, ***Self-efficacy, Stress***, Anxiety***, Burnout, Mental Resources, Work ability,*** Mastery orientation, Avoidance orientation |
| - Intentions |  | A prior conscious decision to perform a behavior, often equated with the goals defined by the task instructions | Job search intentions, Retirement intentions, ***Turnover intentions,*** Attendance |
| - Cognitions |  | All forms of knowing and awareness, such as perceiving, conceiving, remembering, reasoning, and imagining | ***Attributions, Goals***, Group/Team., Cognitive focus, Generative thinking, On-task cognition, Decision-making style, Cognitive focus |
|  | - Goals | The end state toward which a person strives: the purpose of an activity or endeavor. | Goal difficulty, Goal clarity, Goal frame, Goal proximity, Assigned goals, Goal content |
|  | - Attributions | An inference regarding the cause of a person’s behavior or an interpersonal event | Stability attributions, Internal attributions, Ability attribution, ***Strategy attribution, Effort attribution, Ability attribution, Circumstances attribution,*** Supervision attribution, Luck attribution |
| * Adapted based on the APA Dictionary of Psychology | | |  |
| ** In bold and italic variables included in the study | | |  |
